# Cellular perspectives for improving mesophyll conductance

**DOI:** 10.1111/tpj.14656

**Published:** 2020-01-23

**Authors:** Marjorie R. Lundgren, Andrew J. Fleming

**Affiliations:** ^1^ Lancaster Environment Centre Lancaster University Lancaster LA1 4YQ UK; ^2^ Department of Animal and Plant Sciences University of Sheffield Western Bank Sheffield S10 2TN UK

**Keywords:** cell division, cell growth, cell wall, leaf, CO_2_ transport, mesophyll conductance

## Abstract

After entering the leaf, CO_2_ faces an intricate pathway to the site of photosynthetic fixation embedded within the chloroplasts. The efficiency of CO_2_ flux is hindered by a number of structural and biochemical barriers which, together, define the ease of flow of the gas within the leaf, termed mesophyll conductance. Previous authors have identified the key elements of this pathway, raising the prospect of engineering the system to improve CO_2_ flux and, thus, to increase leaf photosynthetic efficiency. In this review, we provide a perspective on the potential for improving the individual elements that contribute to this complex parameter. We lay particular emphasis on generation of the cellular architecture of the leaf which sets the initial boundaries of a number of mesophyll conductance parameters, incorporating an overview of the molecular transport processes which have been proposed as major facilitators of CO_2_ flux across structural boundaries along the pathway. The review highlights the research areas where future effort might be invested to increase our fundamental understanding of mesophyll conductance and leaf function and, consequently, to enable translation of these findings to improve the efficiency of crop photosynthesis.

## Significance Statement

Increasing CO_2_ flux has been identified as an element of leaf structure/function which could significantly improve photosynthetic efficiency and, thus, crop yield. This review highlights the challenges and opportunities of achieving this aim, bringing into focus our ignorance of some fundamental aspects of leaf developmental physiology, and identifying areas where future research investment could yield improved knowledge, understanding, and impact.

## The concept of mesophyll conductance

Once it has crossed the leaf epidermis via the stomatal pores, CO_2_ faces a long and intricate path to reach the site of carboxylation, ribulose bisphosphate carboxylase (RuBisCO), buried deep within the factories of photosynthesis, the chloroplast. These factories are themselves trapped within the array of cells that form the leaf mesophyll. Thus, the delivery of a key raw material for the factory (CO_2_) involves the transport of cargo across multiple boundaries and pathways, each of which will inevitably lead to some delay in the transport and, thus, the delivery of CO_2_ to the factory door. Depending on how active the factory currently is (i.e. its requirement for raw materials), these limitations on the flux of CO_2_ may restrict the ability of the factory to make the finished products (i.e. three‐carbon sugars) upon which the cell and, indeed, the plant depend. This sequence of barriers to CO_2_ movement can be viewed as a series of resistances within the leaf, which (as an inverse) define a conductance to CO_2_, termed mesophyll conductance, *g*_m_. The role of *g*_m_ in limiting the efficiency of photosynthesis has been extensively discussed (Evans *et al.*, 2009; Kaldenhoff, 2012; Tholen *et al.*, 2012; Evans, 2013; Ren *et al.*, 2019), with the current consensus suggesting that improvements to *g*_m_ could increase overall photosynthetic efficiency on the order of 5–10% (Zhu *et al.*, 2010). Consequently, some studies have set out both to accurately characterize *g*_m_ and, concomitantly, attempt to improve the parameter with a view to enhancing photosynthesis (Uehlein *et al.*, 2003; Ellsworth *et al.*, 2018).

The actual estimation of *g*_m_ is experimentally somewhat fraught. *g*_m_ is influenced by a combination of anatomical, biochemical, and environmental factors (Heckwolf *et al.*, 2011; Terashima *et al.*, 2011; Flexas *et al.*, 2012) and assigning values along the various borders of resistance to CO_2_ flow is not trivial. Although a number of methods to estimate *g*_m_ have been established (Table 1; Flexas *et al.*, 2008) each method involves assumptions or limitations, reducing the accuracy, reliability, and repeatability of the method. Indeed, standard deviations in measurements of *g*_m_ can reach nearly 40% of the mean value (Warren, 2006) making it difficult to identify small differences in this parameter. Four key methods to estimate *g*_m_ have been established: chlorophyll fluorescence coupled to gas exchange (Harley *et al.*, 1992); carbon isotope discrimination coupled to gas exchange (Evans *et al.*, 1986; Sharkey *et al.*, 1991; Loreto *et al.*, 1992; Tazoe *et al.*, 2011); oxygen isotope discrimination methods (Barbour *et al.*, 2016); A‐C_i_ curve fitting (Ethier and Livingston, 2004); and leaf anatomy analysis (Niinemets and Reichstein, 2003; Tosens *et al.*, 2016; Han *et al.*, 2018). Each of these approaches has its own limitations (which have been discussed elsewhere, for example Flexas *et al.*, 2013), and the reader is referred to these articles for more detailed analysis. This difficulty in reliably and accurately estimating *g*_m_ raises the question ‘How do you show you have ‘improved’ something when there is some doubt over how to accurately quantify it?’ While the methods that are currently available to estimate *g*_m_ provide reasonable assessments of the relative importance of different aspects of the trait, we would tend to caution on stressing or comparing absolute values calculated by different methods. They can be used as a guide to indicate improvements (or failures), with the ultimate test to determine improvements to *g*_m_ being whether any change has led to the expected shift in photosynthesis.

**Table 1:** Methods used to estimate mesophyll conductance

Method	Reference
Single point online carbon isotope discrimination coupled to gas exchange	Evans *et al*. (1986), Sharkey *et al*. (1991), Loreto *et al*. (1992)
Slope‐based carbon isotope discrimination coupled to gas exchange	Evans *et al*. (1986), Voncaemmerer and Evans (1991), Lloyd *et al*. (1992)
Constant J – chlorophyll fluorescence coupled to gas exchange	Bongi and Loreto (1989), Harley *et al*. (1992)
Variable J – chlorophyll fluorescence coupled to gas exchange	Dimarco *et al*. (1990), Harley *et al*. (1992), Epron *et al*. (1995), Laisk *et al*. (2002)
Initial slope of the A‐Ci relationship	Evans (1983), Evans and Terashima (1988)
Gas exchange/recently synthesized sugars	Brugnoli and Lauteri (1991), Lauteri *et al*. (1997)
Real versus apparent compensation point	Peisker and Apel (2001)
A‐Ci curve fitting	Ethier and Livingston (2004), Sharkey *et al*. (2007), Sharkey (2016)
Gas exchange/oxygen isotopes	Barbour *et al*. (2016), Gauthier *et al*. (2018), Ogee *et al*. (2018)
Oxygen sensitivity of photosynthesis	Bunce (2009)
Leaf anatomy	Niinemets and Reichstein (2003), Tosens *et al*. (2016), Han *et al*. (2018)

Irrespective of these challenges, the concept of *g*_m_ is useful since it provides a framework into which different leaf components (both structural and biochemical) can be incorporated and assessed for their relative role in facilitating or blocking CO_2_ flux. Taking this approach, in this review we provide an assessment of the potential for improving *g*_m_ by selection or manipulation of these different components. We lay particular emphasis on the role of leaf cellular architecture (where recent advances have been made), but also consider aspects of the molecular transport processes proposed to play an important role in limiting or allowing flux across the boundaries that lie between CO_2_ as it enters the leaf and its final destination, RuBisCO.

## Improving mesophyll conductance

Numerous excellent articles have considered *g*_m_, identifying a subset of features that are most likely to limit CO_2_ flux within the leaf and which are, consequently, key targets for improving this parameter (Evans *et al.*, 2009; Terashima *et al.*, 2011; Tholen *et al.*, 2012; Gago *et al.*, 2019). Rather than regurgitate past discussions, we take these previously identified features as the starting point for our review. This will take the form of a consideration of the extent of our understanding of the factors that influence the parameter and, consequently, an estimate of our present and future ability to manipulate and improve *g*_m_ via manipulation of these parameters.

### Exposed mesophyll surface area (*S*_mes_)

At ‘birth’ all plant cells are fixed to their parent by a cell wall. This is derived from the cell plate, which arises from the phragmoplast, whose position is itself determined (in a still somewhat mysterious fashion) by the pre‐pro‐phase band of microtubules during a very early phase of cytokinesis (Smertenko *et al.*, 2017; Facette *et al.*, 2019). If cell division simply followed on untrammelled, then plants would consist of a solid body of tissue without any internal airspace. Beautiful real‐time imaging of plant embryos reveals that this is indeed the structure of a plant during the earliest stages of development (Bassel *et al.*, 2014). However, after germination, as the leaves initiated from the shoot apical meristem grow and differentiate, small air spaces appear at the interstices (Esau, 1967; Pyke *et al.*, 1991), leading eventually to a mature leaf histology comprising distinct cell types (epidermis, mesophyll, vasculature) that are defined not simply by relative position but also by relative cell size, shape, and the degree of airspace between them.

**Figure 1:**
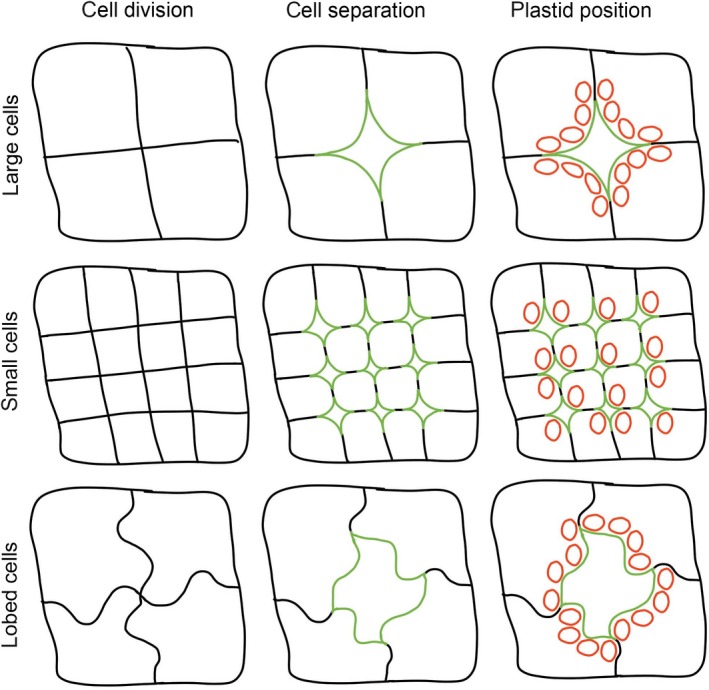
The influence of cell size, shape and separation on potential and actual mesophyll conductance. After cell division to generate a theoretical field of tissue comprising large cells, small cells or lobed cells (first column), the potential exposed surface area for gas exchange is defined by the total length of cell–cell contact (black lines). The actual exposed surface area (green lines in the second and third column of figure parts) depends on the degree of cell separation that occurs. If each cell undergoes an equivalent relative degree of separation, the amount of exposed surface area is higher in both small‐celled and lobed‐cell tissue relative to the large cell tissue. Consequently, plastids (red in the third column) can align so that in the small‐celled and lobed‐cell variants, virtually all plastids gain good access to the exposed surface area of the mesophyll cells (green) across which CO_2_ must flow. In contrast, if plastid number and size is constant, at least some of the plastids in the large cell tissue have difficulty fully accessing the exposed surface area. The ‘excess’ exposed mesophyll surface area in the small‐celled variant is such that even if the plastid number was doubled, most of the plastids would gain access to exposed mesophyll surface area, whereas a similar increase in the large–celled variant would lead to a large proportion of plastids not gaining easy access to the exposed cell surface area, with the lobed‐cell variant having an intermediate phenotype.

Focusing on the mesophyll (the bulk of the ‘middle’ cells lying between the upper and lower epidermis and which play a primary role in photosynthesis), the final value of *S*_mes_ depends on a number of factors (Figure 1):

- The number of cells per tissue volume. If there are more cells per volume, there is more cell surface area per volume, thus the *potential* exposed surface area per tissue volume increases.
- The size and shape of cells. If the cell surface area to cell volume ratio increases, then, clearly, the *potential* exposed cell surface area per volume increases.
- The *actual* degree of cell separation. The exposed surface area per volume achieved from the *potential* *S*_mes_ (determined by (i) and (ii)) depends on the extent to which separation actually occurs along joining cell edges. Clearly not all mesophyll cell surface can be exposed to air – there will be a minimum level of connection required to prevent physical collapse – but what is the optimum or minimum needed to ensure sufficient flux of CO_2_ for the photosynthetic machinery? In the following sections these points are considered individually, although in reality they are highly integrated.

#### Control of mesophyll cell size

Final size is determined by the initial cell size at formation (following division of the mother cell), the rate of subsequent growth, and the duration of growth. In plants, the control of final cell size is complicated by the fact that, although during the initial phase of growth the increase in size may be accompanied by cell division, for most plant cells, the majority of growth occurs once cell division has ceased (i.e. cell division‐independent growth). This is distinct from many other eukaryotic systems used to examine growth phenomena, where termination of cell division is generally linked to cessation of growth. Thus, although cell division and growth are linked in plants, they are mechanistically distinct, with cell division‐independent growth primarily involving extensive vacuolar enlargement and associated expansion/synthesis of the plasma membrane and cell wall material enclosing the cellular material. Although cytoplasmic volume increases, it does not scale with growth in the way it does during cell division‐dependent growth observed in, for example, meristems.

If cell division‐independent growth is key to determining the final size of a mesophyll cell, what is the mechanism controlling this process? A large body of evidence indicates that the structure of the cell wall ultimately sets the boundaries conditions for growth (Ali and Traas, 2016; Chebli and Geitmann, 2017; Cosgrove, 2018). Viable plant cells maintain a relatively high internal hydrostatic pressure (turgor) that is contained by the mechanical properties of the wall surrounding those cells. This wall is a highly dynamic and flexible structure whose properties can be temporally and spatially modulated to constrain or permit growth. The rate of cell growth, therefore, is largely set by the mechanical properties of the wall and, importantly, termination of growth and the setting of final mesophyll cell size, will also be influenced by the cell wall. Unfortunately, how a cell ‘knows’ that it has reached the ‘correct’ final size is one of those fundamental questions in biology that remain surprisingly unclear and contested (Ginzberg *et al.*, 2015). Progress is being made in plant systems (Serrano‐Mislata *et al.*, 2015; Jones *et al.*, 2017), but our understanding remains limited. Similarly, our knowledge of the structure and function of the mesophyll cell wall and growth in model plants, such as Arabidopsis, let alone in crop species, is very incomplete. The majority of research on cell wall structure/function has focused on other tissues, so the transposition of this knowledge to mesophyll cells requires a slight leap of faith. Nevertheless, given the conservation of basic aspects of cell wall structure, they are most probably indicative of the types of genes and encoded activities involved in regulating mesophyll cell growth and size. For example, work on a range of tissues has highlighted the role of pectins (polymers based on galacturonic acid) in modulating cell wall properties and, thus, growth (Peaucelle *et al.*, 2012; Braybrook and Jonsson, 2016). These polymers are synthesized and delivered to the wall in a methyl‐esterified form, with the pattern and degree of methylation subsequently modified by an array of pectin methylesterases (PMEs) whose activity can itself be subject to control by a series of pectin methylesterase inhibitors (PMEIs). Partial demethylesterification can lead to stiffening of the cell wall by permitting calcium cross‐linking between adjacent pectin chains, whereas more extensive demethylesterification can allow pectate lyase enzymes to access and cleave the backbone of the molecule, causing mechanical softening through pectin breakdown. Theoretically, targeted expression of PMEs and PMEIs could be used to modulate the properties of mesophyll cell walls and, consequently, modulate the rate and extent of growth. However, although it is highly plausible that modulation of pectin plays a role in modulating mesophyll cell growth and final size, there are little hard data to support this proposition. Similarly, although there are some data implicating other cell wall proteins in leaf growth, most notably expansins (Cosgrove, 2000), the evidence is mixed on the generality of the phenotypes observed, with the evidence suggesting that expansin efficacy depends to a large extent on the developmental state of the cell wall (Sloan *et al.*, 2009).

These observations highlight a major issue in this area; we still lack a clear consensus on which aspects of cell wall architecture are actually the most important with respect to regulation of structural properties determining or limiting cell growth. A recent revisiting of ideas on cell wall structure/function has begun to produce new ideas on where load bearing occurs within the wall and, consequently, the key points for potential regulation of cell wall mechanical function. For example, one established view is that short hemicellulose chains interact with cellulose through non‐covalent hydrogen bonding, thereby tethering adjacent microfibrils. These polysaccharides are therefore anticipated to play an important role in cell wall loosening for growth. However, a mutant lacking detectable levels of the primary eudicot hemicellulose xyloglucan displayed only a slight reduction in overall growth, despite being mechanically compromised in other respects, suggesting that other cell wall components must also be able to facilitate controlled cell expansion (Cavalier *et al.*, 2008). Furthermore, modelling work suggests that hemicellulose tethering of cellulose fibrils alone will not provide adequate mechanical strength to maintain wall integrity during growth (Yi and Puri, 2012). In addition, recent work has suggested that direct microfibril interactions occur to form a cellulose network which could be a source of the strength that hemicelluloses appear unable to provide (Zhang *et al.*, 2017). Finally, evidence from solid‐state nuclear magnetic resonance (NMR) spectroscopy also suggests that pectin interacts with cellulose directly, a result not observed in previous investigations based on *in vitro* binding assays (Wang *et al.*, 2015).

Clearly, despite many decades of research, our understanding of the spatial and dynamic interactions that occur between polymers in the plant cell wall is still surprisingly open to debate. At present, although we know that modulation of cell wall properties must be the key to regulating mesophyll cells size (and thus potentially influencing *g*_m_), we have very little idea of which of the myriad genes (and combinations thereof) encoding wall modifying enzymes should be the target for manipulation.

An alternative is to take a purely genetic approach, screening for leaves from mutant populations that have larger or smaller mesophyll cells. This has been successfully done in Arabidopsis, revealing that it is possible to generate leaves of similar dimensions but comprising cells of distinctly different average sizes (Horiguchi *et al.*, 2006). Despite identification of the genes involved, the cell wall modifying enzymes or cell cycle products that might have been predicted remain unknown (Kim *et al.*, 1998; Kim *et al.*, 2002). Although making the mechanistic link of how these gene products actually alter mesophyll cell size is sometimes challenging, they nevertheless provide a genetic resource that can be explored to test hypotheses on the link between cell size and *g*_m_. Obviously the potential pleiotropic effects of these mutations (e.g. Yano and Terashima, 2001) require some caution in interpretation of data, but further exploration of these resources from a perspective of photosynthetic biology would be informative.

#### Control of mesophyll cell division

The cell cycle in plants follows the highly conserved format found in all eukaryotes, with slight variations mainly reflecting the particular challenges involved in cytokinesis in a stiff, walled cell. Various reviews provide insight into the intricate details of the machinery involved in both replicating nuclear DNA and separating the products appropriately to generate two viable daughter cells (De Veylder *et al.*, 2007; Polyn *et al.*, 2015). Briefly, the cycle consists of four phases (G1, S, G2 and M), with transitions between the phases being driven by highly conserved cyclin‐dependent kinases (CDKs) whose activity is regulated in both a positive and negative fashion by a series cyclins and CDK‐inhibitor proteins. In some circumstances M phase can be truncated so that a cell undergoes repeated cycles of DNA replication without cell division, a process known as endoreduplication, generating polyploid cells (De Veylder *et al.*, 2011). This is a common occurrence in plant tissues and is of relevance here since increase in ploidy level is often associated with an increase in cell size (Sablowski and Carnier Dornelas, 2014). This correlation is, however, not absolute, with examples of cells within Arabidopsis leaves attaining distinct ploidy level without any apparent shift in size (Autran *et al.*, 2002).

Transgenic experiments over many years have demonstrated that manipulation of genes encoding cell cycle regulators can be used to generate leaves in which cell size is altered, providing useful tools to test hypotheses on the relationship of cell size to *S*_mes_ (De Veylder *et al.*, 2001; Wyrzykowska *et al.*, 2002; Dewitte *et al.*, 2003; De Veylder *et al.*, 2007). Again it must be emphasized that although the number of cells per tissue volume sets the potential for *S*_mes_, the realization of this potential depends on other, downstream factors. Exactly how the absolute value of final cell size is set in transgenics in which cell cycle regulators have been mis‐expressed remains unclear, but the empirical observation is that manipulations that promote the cell cycle tend to lead to leaves with smaller cells, and manipulations that repress the cell cycle lead to leaves with larger cells. Presumably, prolonged expression of positive regulators of the cell cycle leads to an extension of the cell‐division‐dependent phase of development, so that more cells are generated within a specific time phase. Why the ‘extra’ cells generated do not proceed to attain the ‘normal’ size observed in non‐transgenics, however, remains unknown. It appears that there is a supracellular level of control of organ size so that even if more constituent cells are generated, this does not necessarily lead to increased final organ size (i.e. mesophyll cell size is subservient to a more global internal regulator of growth). This frequently observed intransigence of organ level size to modulation of constituent cellular components is termed compensation, the molecular mechanism of which also remains unclear (Hisanaga *et al.*, 2015). Conversely, in the situation where the cell cycle is inhibited, growth appears to go beyond the ‘normal’ check point at which cell division occurs, leading to termination of cell growth at a larger set point than observed in non‐transgenic plants. Again, a compensation phenomenon is often observed so that, although some reduction in leaf size is generally observed, the final outcome on leaf size is not as great as might be expected. As indicated earlier, although there are various theories on how cell cycle regulation is linked to cell size (Ginzberg *et al.*, 2015; Schmoller *et al.*, 2015), this remains a contested area in biology. Therefore, with respect to understanding the control of mesophyll cell size, at present we are limited to empirical data indicating that altered regulation of specific cell cycle regulators can be used to influence final cell size, with the precise mechanism awaiting elucidation. Nevertheless, the tools available to alter the cell cycle do provide a way in to (indirectly) alter mesophyll cell size and, thus, a means to explore the link to *g*_m_.

#### Control of mesophyll cell shape

During cytokinesis a new cell plate is formed, dividing the mother cell into two daughter cells. Simple observation shows that cell shapes in the mesophyll tend to be highly regular and repeated (exemplified by mesophyll cells in grass leaves and the palisade cells in eudicot leaves, but note the more varied shapes displayed in the eudicot spongy mesophyll) (Esau, 1967). Although differential growth can alter cell shape subsequent to division, the initial geometry of a cell at ‘birth’ will generally restrict its future shape trajectory.

At a global, organ level, we have a good idea of the spatial patterning of transcription factors and the exchange of signals during early development that leads to the determination of the fundamental adaxial and abaxial domains of the leaf primordium (Bar and Ori, 2014). Disruption of these early patterning processes leads to major shifts in whole leaf morphology and altered constituent cell shape and size. However, exactly how these initial domains of transcription factor expression become transduced into the ordered (or less ordered) patterns of cell division that play such a major role in defining mesophyll cell shape remains very unclear. Similarly, at the level of the individual cell, we have a good phenomenological description of the events that led to oriented cell plate formation, but our understanding of the underpinning molecular processes are more limited (Smertenko *et al.*, 2017). Thus, during the G2 phase of the cell cycle, microtubules and actin assemble to form a pre‐prophase band (PPB), which marks the position at which the newly formed cell plate will align later in mitosis to set the position of the nascent cell wall, dividing the mother into two daughter cells. Depending on the orientation of the division plane and its relative symmetric or asymmetric position within the mother cell, the PPB will determine both the initial shape and size of the two daughter cells. Markers of the PPB position have been identified (Walker and Smith, 2002; Walker *et al.*, 2007; Lipka *et al.*, 2014) with loss of expression of the gene encoding these markers (e.g. *TANGLED*) leading to leaves with numerous abnormal cell division planes and, thus, abnormal mesophyll cell shapes. How the plane of orientation of the PPB is controlled remains contested, with various ideas and models suggesting, for example, shortest cell wall splitting a cell, minimal energy configurations, etc. (Besson and Dumais, 2011; Yoshida *et al.*, 2014; Louveaux *et al.*, 2016). How these rules are configured in a situation in which new cell walls do not take up obviously minimal energy or shortest cell lengths is open to speculation. The data suggest a supracellular vectorial system imparting growth polarity across portions of the leaf, but the molecular nature of those vectorial signals remains unknown (Abley *et al.*, 2013).

Once the new cell is formed, subsequent growth of the cell can be isotropic (the cell increases in size, but the essential shape remains the same) or anisotropic (there is differential growth along different cell axes so that in addition to becoming larger the cell shape changes) (Figure 1). This anisotropic growth can be at the scale of the whole cell (principle axes of cell growth) or at a local scale within the cell (leading to local shifts in shape, for example lobes). At the cell scale, the principle growth direction is widely accepted to be determined by the alignment of the inextensible cellulose microfibrils, which constrain growth overall, but permit growth in the perpendicular direction (Suslov and Verbelen, 2006). As with the general principles of cell expansion described above, cell shape changes ultimately depend upon the cell wall structure, but in this context, it occurs at a local wall level to create anisotropy within the cell, defining how the wall responds to uniform turgor pressure. Thus, the potential targets for altering growth vectors within mesophyll cells are similar to those involved in overall size control, but the question becomes one of how the activity of wall synthesis/modifying enzymes is locally modulated along the main axes of a cell to locally modulate cell wall mechanical properties. For example, it has been demonstrated that asymmetric pectin modification in the hypocotyl is required for anisotropic growth (Peaucelle *et al.*, 2015). Expansins may act locally, with their action depending on local cell wall properties, such as the distribution of xyloglucan‐rich ‘hotspots’, so local modification of cell wall architecture may dictate where more generally expressed wall loosening factors can act to release an inherent anisotropy within the cell (Wang *et al.*, 2013). This, of course, simply pushes the question back a step as to how such inherent anisotropies in wall structure are set up in the first place. It has been shown that a plant‐specific Rab protein is required to specify geometric cell edges in young organ primordia (Kirchhelle *et al.*, 2016), so vesicle‐mediated delivery of cell wall material can act to influence stiffness at the cell edges.

At the subcellular scale, there has been a focus on lobes as an effective means to increase exposed mesophyll cell area for CO_2_ uptake, with a high degree of lobing being linked to high *g*_m_ (Sage and Sage, 2009). The formation of lobes in plant cells has been most intensively studied in the leaf epidermis where intricate jig‐saw puzzle forms are common and which, due to their position on the leaf surface, are relatively easy to visualize (Carter *et al.*, 2017; Sapala *et al.*, 2019). A series of elegant papers has revealed changes in the cytoskeleton (tubulin/actin) at the neck of lobes, with differential distribution of cell wall epitopes along cell perimeter being produced, which set up local gradients of stress/strain along the cell wall perimeter, resulting in local outgrowth (lobes) (Sampathkumar *et al.*, 2014). There is continuing discussion as to what extent cytoskeletal patterns initiate perimeter pattern or reinforce pattern that is already pre‐set. One possibility is that the local cell wall patterns and actual length of perimeter set the scene for a buckling of the system, leading to the observed geometric patterns (Bidhendi *et al.*, 2019). Presumably similar molecular mechanisms underpin the control of mesophyll cell shape.

At present, the limited tools available for targeted direct manipulation of cell shape make engineering this parameter in the mesophyll a challenging task. As with cell size, a more tractable approach may simply be to perform large‐scale genetic screens to identify mutants with altered cell shape, then to analyze these for photosynthetic performance, for example *g*_m_. The challenge here is that mesophyll cells are, by definition, not exposed to surface imaging, so analysis requires the use of more advanced imaging techniques (Earles *et al.*, 2019) and more extensive tissue processing, making large‐scale phenotyping more challenging. Despite these issues, increased efforts to screen for mesophyll cell shape (and size) variants would be a good pathway to explore functional relationships to *g*_m_.

#### Control of mesophyll cell separation

Although it may be possible to generate a block of mesophyll with relatively small, highly lobed cells with a large potential for enhanced *S*_mes_, this potential will only become reality if cell separation occurs (Figure 1). However, the majority of research has focused on characterizing wall components and how these components fit together in a 3D matrix (Park and Cosgrove, 2012; Zhang *et al.*, 2016; Anderson, 2018). Analysis of cell wall degradation in recent years has focused more on the identification and development of enzymatic tools to enable biotechnological use of plant material as a renewable energy source. This had led to an increased palette of cell wall modifying activities aimed at providing more efficient depolymerization of cell wall polysaccharides for the generation of substrates suitable for fermentation (McCann and Carpita, 2008; King *et al.*, 2011). By their very nature these efforts generally provide limited information on cell wall separation at the cellular resolution of the leaf mesophyll. Probably the clearest insight from these efforts is the degree to which cell wall material from different sources can require distinct combinations of enzymes to allow degradation, reflecting the diversity in cell wall composition. With respect to mesophyll cell separation, these data serve to remind that although some general principles of the process can hopefully be elucidated, each plant system may have its own variation depending on subtle differences in cell wall composition and structure.

When looking at our understanding of endogenous enzymes involved in plant cell separation, significant recent advances have come from analysis of abscission (Lee *et al.*, 2018; Lee, 2019). This has revealed a syncopated exchange of local signals (reactive oxygen‐based) between cells, leading to the formation of localized secondary cell wall synthesis, which prepares the future break point for exposure. However, clearly the leaf mesophyll does not normally form lignin and, in contrast with abscission, the degree of cell separation is only ever partial. More insight may come from recent advances in our understanding of lateral root development whereby cells overlying the emerging lateral root separate to allow the new organ to emerge (Kumpf *et al.*, 2013). A swathe of genes encoding cell wall modifying enzymes has been identified that presumably play a role in the separation events in the root cortex. However, the functional role of individual enzymes remains to be tested, and the relevance of the root‐based system in which a physical force generated by the lateral organ helps to push cells aside to what happens in the leaf mesophyll, where pairs of cells separate without an obvious source of external force, is open to speculation. Perhaps the most insightful data for understanding the mesophyll comes from experiments on differentiating stomata (Rui *et al.*, 2017). In the final step of guard mother cell differentiation, the middle portion of the wall between the two nascent guard cells separates to form the pore required for gas exchange from the atmosphere into the internal mesophyll. These data indicate a role for polygalacturonase, that is localized pectin degradation, in partial wall digestion followed by partial cell separation. Obviously stomata are positioned very close to the mesophyll cells where a similar partial cell separation must occur in a co‐ordinated fashion to create the air channels through the leaf by which CO_2_ accesses the more internal mesophyll cells (Lundgren *et al.*, 2019). It seems plausible that a similar but repeated spatiotemporal process of localized pectin breakdown is involved in this regulated process of mesophyll cell separation. Data are still missing to support this speculation, but identification of genes encoding pectin modifying enzymes that are expressed in the appropriate pattern to elicit these changes in mesophyll separation might be a productive route of research to understand the process of mesophyll cell separation and, as a consequence, provide tools to modulate the process.

### The cell wall and mesophyll conductance

Having created an interface by which CO_2_ can move from the intercellular airspace into the surrounding tissue, there are a number of physical obstacles to the free flow of gas to the site of carboxylation within the chloroplast. Most importantly, the pathway now shifts to an aqueous environment and CO_2_ flux through water provides a much higher resistance to flux than in air (Evans *et al.*, 2009). Secondly, there are polymer‐based barriers in the form of the carbohydrate‐based cell wall and the lipid‐based membranes (plasma membrane and two chloroplast membranes). How does CO_2_ traverse these barriers and what is the potential of increasing conductance across them?

Starting with the primary cell wall, it is essentially a water‐saturated gel comprised predominantly of carbohydrate polymers. Some of these polymers will be charged, but is highly unlikely that ionic interactions occur with non‐polar CO_2_ to brake the flux of the gas (Terashima *et al.*, 2011). At a larger scale, water is expected to move freely across the cell wall (Kramer *et al.*, 2007), so the main negative outcome of the cell wall on CO_2_ flux may be simply that because the cell wall components take up space, the volume of free water for CO_2_ diffusion is decreased in the wall (Tomas *et al.*, 2013). Any secondary cell wall modifications (e.g. lignification) would act to restrict hydration and, thus, CO_2_ flux, but such secondary cell modifications are generally not observed across the exposed surfaces of mesophyll cells via which CO_2_ flux occurs. The network of wall polymers will provide a degree of steric hindrance, but generally it is thought that the actual thickness of the cell wall has the most influence on CO_2_ flux (Tomas *et al.*, 2013; Gago *et al.*, 2019). However, the situation may not be simple. For example, a rice mutant with thinner cell walls had lower *g*_m_, which was interpreted as a reflection of greater tortuosity of the CO_2_ pathway within the cell wall. Measurements of the CO_2_ permeability of the cell wall are urgently required. Irrespective of the mechanism by which a lower *g*_m_ was achieved, simply making walls thinner might not be a direct route to improving *g*_m_ (Ellsworth *et al.*, 2018). There are, of course, considerations about just how thin a mesophyll cell wall can be. Primary cell walls already tend to be relatively thin (four or five layers of cellulose fibrils), raising the question of whether such walls can be made thinner without compromising structural integrity (Carpita and Gibeaut, 1993; Cosgrove, 2018). The answer will probably be plant specific, depending on the size of the mesophyll cells and the thickness/composition of the cell walls that support/contain the cells. There is also a link here to the degree of cell separation. When cells remain joined by a shared wall, the turgor pressure generated within each cell tends to cancel out the other so that the overall resultant tensile force in the joining wall may be low. When cells separate so that a portion of cell wall becomes exposed to intercellular airspace, that portion of wall will have a tendency to bulge out, and consequently contain a higher tensile stress, which may necessitate altered composition and/or thickness of the cell wall. This may automatically decrease flux of CO_2_ across that wall. Once again, our lack of fundamental knowledge of what controls mesophyll cell wall thickness and/or the arrangement of wall polymers, makes targeted changes of mesophyll cell wall thickness to improve *g*_m_ challenging. It is also worth noting the recent proposal that cell lobing is dependent on the distribution of mechanical properties around the cell perimeter (Bidhendi *et al.*, 2019). Changes that alter cell wall thickness in a uniform fashion might act to disrupt local gradients in wall properties important for lobe initiation, and thus have a negative outcome on cell shape parameters that are important for promoting CO_2_ conductance.

### Membrane‐based facilitation of CO_2_ flux

CO_2_ is non‐polar and hydrophobic, thus one might expect lipid‐based membranes to pose only a limited barrier to its diffusion (Endeward *et al.*, 2017). However, a swathe of experimental data (from both the plant and animal fields) suggests otherwise, leading to the identification of a family of transporters (termed aquaporins due to their initial characterization with a role in water transport) as potentially facilitating CO_2_ diffusion across membranes (Kaldenhoff, 2012). In plants, knock‐down and knock‐out data support the proposal that aquaporins play a physiological role in CO_2_ transport and that increased CO_2_ flux (increased *g*_m_) can be brought about by overexpression of these transporters (Uehlein *et al.*, 2003; Flexas *et al.*, 2006; Uehlein *et al.*, 2008; Heckwolf *et al.*, 2011). The importance of this flux remains somewhat debatable (Kromdijk *et al*., 2020), with results from the animal field suggesting that the influence of aquaporins on CO_2_ flux may be highly context dependent. For example, the relative lipid composition of the membranes can have a major influence on the basal flux of CO_2_ across the membrane, so that the outcome of any potential increase in CO_2_ flux may depend upon the lipid composition of the membrane into which the transporter is inserted (Endeward *et al.*, 2017). Moreover, it is clear that many membranes are protein‐rich structures in which the ‘free’ bilipid area available for CO_2_ diffusion may actually be quite limited, amplifying the potential benefit of inserting extra CO_2_ transporters in to those membranes. The complex organization of, for example, thylakoid membranes exemplifies the structural dynamics at play (Ruban and Johnson, 2015), suggesting that further investigation of chloroplast envelope membranes and associated transporters are well warranted. The data in this area for CO_2_ transport are limited and complex. For example, although initial plant aquaporins were identified as specifically localized to the plasma membrane, later data showed that at least a portion of the protein was found in the inner chloroplast membrane, and it was the plastid membrane that showed the highest increase in CO_2_ conductance after manipulation (Uehlein *et al.*, 2008). This suggests a situation where one gene can express a protein that ends up in two locations, with the effectiveness of the transporter being dependent on the final location. Added to the relatively large gene family encoding aquaporins in plants, further dissection of the roles of different CO_2_ transporters remains to be elucidated.

To summarize, although the role of endogenous aquaporin‐mediated CO_2_ flux may be highly context dependent and complex, it does appear that endogenous membrane systems (particularly in protein dense systems typified by those found in chloroplasts) have a lower endogenous CO_2_ flux than might be expected from consideration as a simple lipid bilayer. Targeting increased CO_2_ permeability to these membranes should lead to a general increase in *g*_m_ and is a tractable approach, providing that the level of overexpression of the transporters does not itself lead to disruption of normal protein dynamics within the plastid membranes required for chloroplast function.

### The dynamics of mesophyll conductance

Most of the work on *g*_m_ considers the system at steady state. With respect to leaf cellular architecture, this is reasonable since structural aspects of, for example, exposed mesophyll area or cell wall thickness, are unlikely to change rapidly once the leaf has differentiated (though of course it will change during early phases of development). However, other more biochemical‐based features that may influence CO_2_ flux (e.g. CO_2_ channels) can vary much more rapidly, changing in response to, for example, diel or environmental factors. This raises another potential layer of complexity, with few studies aiming to measure *g*_m_ over the time scales relating to the rapidity at which gene expression might alter the level of transport proteins (min/h). The studies that have considered variation of *g*_m_ over time have reported shifts indicating that the system can respond and accommodate to varying conditions to adjust CO_2_ flux accordingly (Flexas *et al.*, 2007), and a response of *g*_m_ to shifts in temperature has also been reported (Scafaro *et al.*, 2011). These data indicate that there are endogenous regulatory systems that can modulate *g*_m_ (by mechanisms that remain unknown). This raises the caution that efforts to improve *g*_m_ may be thwarted by autoregulatory systems, which might tend to restore *g*_m_ to an endogenously set‐level. The nature of this autoregulation, and how the ‘set’ level is ordained, remains very unclear at a molecular level, yet such knowledge may be a pre‐requisite for engineering the system.

The other level of dynamics in the system is at the scale of the organelle. Chloroplasts are mobile within the cell and can also change shape. From theoretical considerations, to minimize the flux pathway of CO_2_, chloroplasts should be arranged close to the plasma membrane in locations where the mesophyll cell wall is exposed to the intercellular airspace (Figure 1). This leads to a maximal value of *S*_c_ (exposed chloroplast membrane for CO_2_ uptake) per exposed area of mesophyll cell wall (*S*_mes_). The ratio *S*_c_/*S*_mes_ has been identified in numerous studies as an important determinant of *g*_m_ (Evans, 2013; Tomas *et al.*, 2013; Gago *et al.*, 2019). Localization of plastids to the cell periphery is generally observed, and movement in response to irradiance at various wavelengths well‐documented. The ability to optimize *S*_c_/*S*_mes_ will depend on our ability to control the number and size of plastids in a cell, and how this is co‐ordinated, not only with cell size/shape but with *S*_mes_ (discussed above).

A number of mutants in chloroplast division machinery have been identified, although they generally lead to fewer, larger plastids, which is likely to decrease the ability of a cell to optimize the spatial distribution of its photosynthetic machinery for CO_2_ uptake (Chen *et al.*, 2018). Although the promotion of chloroplast differentiation can be engineered (Wang *et al.*, 2017), quantitatively manipulating chloroplast number and size in mesophyll cells remains a major challenge (Hymus *et al.*, 2013). A simpler route to increasing the number of chloroplasts per cell (and thus *S*_c_) may be to decrease mesophyll cell size (see previous section). Natural selection may have already taken this route with, for example, rice mesophyll cells becoming so small and tightly lobed that chloroplasts are crammed together with very limited ‘chloroplast‐free’ space around the cell perimeter (Sage and Sage, 2009). Such tight packing of chloroplasts may, however, come at a cost. Depending on irradiance level, chloroplasts have the ability to alter their position to either maximize or limit light capture, which may be of especial importance under conditions of high irradiance where excess energy capture has the risk of leading to significant damage to the cell and tissue. To what extent and when chloroplast density becomes so high that the limited flexibility in movement might offset any advantage in terms of improved *g*_m_ (via higher *S*_c_) is open to speculation. In addition, if a mesophyll cell is packed with chloroplasts and the entire mesophyll cell surface cannot be exposed to intercellular airspace, then some plastids must lie in non‐optimal positions with respect to CO_2_ pathway length. Whether movement of chloroplasts is required to optimize CO_2_ flux at a cell level is unclear, let alone whether this trait can be selected for or engineered. Evidence in support comes from the analysis of mutants with altered chloroplast arrangement (Tholen *et al.*, 2008) and, indirectly, via the observation that plants in which cytoskeletal movement proteins were engineered to increase cytoplasmic streaming had larger cells and improved growth (Tominaga *et al.*, 2013). The influence of this cytoskeletal manipulation on photosynthesis and chloroplast movement would be worth investigating.

The other long‐term dynamic element in the system that needs to be taken in to account is the fact that the atmospheric level of CO_2_ (which drives the diffusion gradient towards the chloroplast) is increasing. Bearing in mind the time taken to generate and breed new crops, we need to be aware that whatever parameter we choose to optimize or modulate today, the plants in 30–40 years will be dealing with significantly higher external CO_2_ concentrations (Ainsworth and Rogers, 2007). Incorporating experimental and modelling approaches to predict the outcome of such changes in atmospheric CO_2_ on *g*_m_ would be advantageous.

### Control of mesophyll airspace

Although gas flux can be decreased when channel diameter becomes very small, clearly the majority of air spaces within leaves are of a dimension far beyond that at which molecular resistance is likely to play a role (Parkhurst, 1994). This raises the question of whether the pattern of airspace observed in leaves is at all limiting to CO_2_ flux and, if not, whether this parameter can be decreased without any adverse effect on CO_2_ flux within the leaf. Experimental data support the case for this to be true, showing that airspace can be essentially be replaced with photosynthetic tissue, leading to a maintained photosynthetic activity on a per tissue volume basis with no decrease in *g*_m_ (Lehmeier *et al.*, 2017_)_. Interestingly, in manipulations in which airspace was filled with small mesophyll cells, there was actually an increase in *g*_m_ (and increased assimilation rate) which correlated with an increased air channel density and smaller air channels (Lehmeier *et al.*, 2017). The mechanistic basis for the link between air channel network parameters (tortuosity, channel diameter) and *g*_m_ is unclear, but it suggests that we may need to revisit our view of CO_2_ flux in the intercellular air space as being something that does not significantly impinge on *g*_m_. It is certainly clear that at least some leaves seem to have more airspace than required simply to allow sufficient CO_2_ flux, and that the pattern of that airspace (an emergent property of cell division, growth and separation events) may not be optimal for photosynthesis. This raises the question: if this trait is advantageous for the leaf, why has evolution/breeding not arrived at this solution? This point links to general comments about potential trade‐offs in *g*_m_ manipulation.

### Trade‐offs

Although the focus in this review has been on the potential for improving *g*_m_, it is highly likely that optimization of this one trait will have knock‐on effects on other desirable traits, that is there will be potential cost involved for potential gain in *g*_m_. For example, if having more, smaller mesophyll cells is a route to improving *g*_m_, then more material will be required to build tissue volume, thus greater carbon, nitrogen, and phosphorous needs. Under agronomic regimes where fertilizer is readily available, this extra cost may not be an issue, but from a sustainability and economic stance, this strategy may be more questionable. There may also be indirect biological costs. For example, although having more, smaller mesophyll cells may be advantageous for photosynthesis on a per area leaf basis, if this leads to smaller leaves there will probably be a negative outcome on light capture at a plant level. A system that is more efficient may nevertheless have a lower overall capacity or output, with knock‐on effects on, for example, total yield. Moreover, while increasing *g*_m_ may improve rates of photosynthesis, it is also associated with greater g_s_ and consequently effects on water‐use efficiency (Tomeo and Rosenthal, 2017). We need to be clear whether it is efficiency or capacity that we are trying to improve via modifying *g*_m_, although in the best‐case scenario one would aim for both.

## Summary and future perspective

The perspectives for improving *g*_m_ described above might seem to be a litany of gloom, providing an extensive list of all the aspects of mesophyll conductance that we either do not know or fully comprehend, limiting our ability to make any coherent plan of how we might improve this parameter. It is clear that without improvements in fundamental knowledge of cell wall structure/function, control of cell size and shape and their integration into leaf form, and even a consensus on what constitutes a ‘good’ way of measuring *g*_m_, efforts to improve *g*_m_ will be hampered. Nevertheless, despite these obstacles, the very concept of *g*_m_ remains very useful. By focusing attention on those aspects of leaf structure and biochemistry that, even at a qualitative level, are most likely to be important for improving *g*_m_, the concept allows a focusing of effort. Continued improvements in screening procedures are providing tools to identify potentially useful traits at a tissue and/or cellular level, which may still prove useful in crop breeding. An improved level of mechanistic understanding would be extremely useful to ensure that efforts are focused most efficiently, but even without this full mechanistic understanding, pathways to the improvement of *g*_m_ can be envisioned. For example, if leaf structural parameters linked to improved *g*_m_ can be identified (Hanba *et al.*, 2004; Han *et al.*, 2018), then with the increasing availability of rapid imaging technologies for screening phenotypic variation (phenomics), new sources of genetic variation could be identified and characterized, providing the raw material for breeding. We may still be some way off ‘designer’ leaves with respect to *g*_m_, but the recent spectacular advances being made with exploring rational design of improved photosynthetic efficiency via modification of biochemical CO_2_ capture pathways are extremely encouraging (South *et al.*, 2019), suggesting that similar approaches are viable to improve mesophyll conductance.

## Conflicts of Interest

The authors declare no conflict of interest.

## Author contributions

MRL and AJF jointly planned and wrote the manuscript.

## Data Availability

The paper does not contain any shared data.
